# Electro Therapeutics

**Published:** 1863-05

**Authors:** H. Lassing

**Affiliations:** 238 Ninth Avenue, New York


ART. II.—*Electro Therapeutics*—By H. Lassing, M. D., *of New York.*

This much neglected, yet valuable therapeutic agent, seems to be but little understood by the profession generally. I am quite accustomed, when suggesting this remedy, to be asked, how many shocks I would give, and similar questions, showing that most commonly electricity is associated with ideas of shocks, pain and indefinite and confused effects. This is not so; these feelings have created prejudices which have paved the way for quacks and mountebanks under the cloak of specialists, to gull the public. People as a general thing look upon electricity with favor, when they are afflicted with any disease they think electricity will cure; they inquire of their physician about it; he, as a general thing, unfortunately, knowing perhaps but little about it, discourages them, and the result most always is, that they are thus stimulated to apply to some quack for an application of the remedy their physician advised them against.

I flatter myself in the belief that I have to some extent succeeded in removing this professional prejudice, by frequent appeals of this kind through the medical press, and reports of the successful application of electricity in various diseases, and would beg the indulgence of your readers, that I may elucidate my ideas of, and experience with electricity.

The form in which electricity is most useful, medicinally, is that of electro-magnetism, which science was created a separate branch by the discoveries of Œrsted, and is based upon the fact that when an electric current, from a single battery, is passed through a long conductor, as a spiral of copper ribbon, or a long wire, it will be found, at the moment of breaking the contact between the conductor and the battery, that vivid sparks will appear, and a feeble shock will be felt, if the moistened fingers grasp the naked conductors, showing that a long conductor supplies the place of an increased number of plates in a voltaic series, and to some degree imparts the quality and intensity to a current of quantity. A secondary current can be induced from this by bringing a long coil of fine, insulated wire within a short distance of the coil already spoken of. By combining and modifying the results thus briefly glanced over a great number of ingenious and beautiful electro-magnetic apparatuses have been produced, founded on these principles, but one of which, however, possesses any value for medical or dental use. To make such an apparatus valuable, for several reasons, some of which are self-evident, and others which I will explain, it must possess a primary or direct current, and this must be sensational. Many possess such a current, but having no intensity, give no sensation, hence is perfectly useless. The only machine I have ever seen in this country, manufactured here, possessing such a current, is that made upon the principle of a patented and very ingenious contrivance by Dr. S. B. Smith, of New York, and I will therefore, briefly describe it A galvanic current from a zinc and platina battery (which are always clean) is made to pass through two different coils of insulated copper wire, some thousands of feet in length, and is broken by an armature. It is furnished in two ways. One, as powerful primary or direct current at one end, and otherwise as a still more powerful to-and-fro or secondary current at the other end of the stand, on which the helix rests. These are the only ways in which electro-magnetism can be made useful to the physician and dentist. Some have even claimed that they furnished six currents. This is too ridiculous an idea to necessitate even a refutation. No matter in what shape you find it, there is but one electro-magnetic current, and this is modified into a strong sensational current, running continually in one direction, owing to the polarity of the induced magnetism, and a stronger sensational current running to-and-fro, owing to the alternate magnetic attraction and repulsion.

The direct current, as proved by Duchenne, has very different properties from the to-and-fro or secondary current, the first being always in one uniform direction; the latter having a to-and-fro direction; the former in many cases, having more influence upon the muscular and nervous systems, the latter upon the skin and internal organs. Much of the value of the current depends upon the number of vibrations of the armature, and for this reason rotary machines are of little or no value medicinally.

Electricity in this form stimulates the heart and arteries to increased muscular contraction, causing an increased flow of blood, having the same effect upon the venous system; thus enabling us rapidly to remove the tendency to venous congestion.

Many have the erroneous impression that an electric current passes through the body as it would through a wire in a very small shape, but experiments with an accurate galvanometer have shown me that an electro-magnetic current, if the primary or direct modification is used, passes in a wave, from the positive to the negative pole, wherever applied to the human body, in the most direct way, along the best conducting tissues, and does not single out the nerves as its conductors, though it can be made to do so by appropriate conductors. This wave averages about twelve inches in width.

The most simple way of applying electro-magnetism is in the case of torpid ulcers, where, through the want of vitality in the part, the ulcer will not heal—in fact, slowly spreads. Nothing is so useful as an application of the direct current through the ulcer, the parts being placed in apposition, and a uniform support given. A new character is given to the flabby granulations, which now spring up red and healthy, and the ulcer quickly heals.

I can only add that I have used electro-magnetism with the greatest success in several cases of paralysis induced by eccentric irritation, infantile and central or cerebral paralysis, neuralgia in its various forms, muscular contractions resulting in deformities, particularly in talipes, slight curvatures of the spine arising from a debility of a muscle or muscles, stiff joints, aphonia, epilepsy, asthma, and many other diseases too varied and multiform to mention, and shall be happy, if acceptable, to give the readers of this Journal an opportunity to judge of the remedy by a synopsis of my method of treatment and reports of cases in some future number.

In conclusion I would say that I do not wish to be understood as advocating electro-magnetism as a cure-all, but that it is a therapeutic agent of the greatest value, and well adapted to very general and successful use.

238 *Ninth Avenue, New York. April*, 1863.

